# Sexual violence in medical students and specialty registrars in Flanders, Belgium: a population survey

**DOI:** 10.1186/s12909-021-02531-z

**Published:** 2021-02-24

**Authors:** M. Geldolf, J. Tijtgat, L. Dewulf, M. Haezeleer, N. Degryse, N. Pouliart, I. Keygnaert

**Affiliations:** 1 Free University Brussels, Brussels, Belgium; 2 Ghent University, Ghent, Belgium; 3 KU Leuven, Leuven, Belgium; 4 University Hospital Brussels, Brussels, Belgium; 5 Ghent University, Dpt Public Health & Primary Care, ICRH, Ghent, Belgium

**Keywords:** Sexual violence, Sexual harassment, Medical students, Bystander actions, Support resources, Flanders, Belgium, Medical school, Medical trainee, Specialty registrars, Graduate, Postgraduate, Undergraduate

## Abstract

**Background:**

Sexual violence has globally been recognized as harmful to young people’s health. In medical school, which is a highly competitive environment, the risk is supposedly even bigger. In this study we firstly aimed to investigate the magnitude and precipitating factors of sexual violence in medical students and specialty registrars in Flanders, Belgium. Secondly, we wanted to assess the reactive behaviours as well as the knowledge of possible types of bystander reactions as well as potential support resources for victims of sexual violence.

**Methods:**

This study was initiated and coordinated by the Flemish medical student representation organisation (VGSO). A survey containing demographic and behaviour-specific questions based on the UNMENAMAIS and SAS-V questionnaire was sent to all undergraduate, graduate and postgraduate students of the 5 medical schools in Flanders. Participants were asked to limit their responses to internship-related events. Further questions concerning reactions to sexual violence, assailants, bystander reactions and general knowledge concerning support after sexual violence were asked.

**Results:**

We received 3015 valid responses to our survey, obtaining a response rate of 29% in the potential target population. Within the total study population, 1168 of 3015 participants (38,73%) reported having been victim of at least one type of sexual violence as explored by our survey. This percentage was the highest in GP specialty registrars (53%), followed by specialty registrars (50%) and master students (39%). Assailants of sexual violence varied, most often they were medical staff members, students or patients. In most types of sexual violence, nobody reacted to this behaviour. Women (57.3%) talked about what happened afterwards more often than men (39.7%). When asked about their knowledge of possible bystander reactions and support services for sexual violence, 60% of the respondents did not know about their existence.

**Conclusions:**

Sexual violence is still a relatively frequent issue in medical students and specialty registrars. Patients form an important part of the assailants. In a third of reported sexual violence cases, nobody reacted. In addition, male victims seem to underreport. There is still much need for sensitisation on support mechanisms and centres for victims and witnesses of sexual violence.

## Background

The World Health Organization (WHO) defines sexual violence as ‘any sexual act that is carried out against someone’s will. It can be carried out by any person, regardless of their relationship to the victim, in any setting’ [1]. Sexual violence is divided in different types according to the degree of physical contact. Recently, sexual violence is grouped in ‘hands-off’ behaviour with different forms of sexual harassment, such as sexual remarks, so called jokes and sexting, and ‘hands-on’ behaviour such as kissing, touching or forced intercourse (sexual abuse and rape) [2, 3].

Reports of sexual violence in medical training date back to the early 90s in the United States [4]. Sexual violence has increasingly been recognized around the world as an issue in medical training and healthcare ever since. Renewed attention was drawn to this issue by the ‘#Me Too’ movement, with broad media coverage of recent revelations of sexual violence, inducing concern about its frequency and impact. Recent studies have shown that between 30 and 50% of specialty registrars self-report an experience of sexual violence during their medical training [5–8].

In Belgium, medical education is divided into three phases. As a bachelor (undergraduate) student, medical students spend most of their time in university halls for theoretical courses. In general, bachelor students are exposed to the hospital environment only during a 1- or 2-week introductory clerkship. After 3 years, students enter their masters (graduate). During the masters (3 years), students participate in at least 12 months of hospital internships in both university hospitals as well as general hospitals and general practitioner’s (GP) offices. Finally, after graduating, students enrol in postgraduate training to become a specialist as specialty registrar (called “arts-specialist in opleiding” or “ASO” in Flanders) for 4–6 years or general practitioner as GP specialty registrar (as “huisarts in opleiding” or “HAIO”) for 3 years. For each internship there is a supervisor who is responsible for the evaluation and education of the intern or specialty registrars.

Several characteristics of medical training programs might predispose medical students and specialty registrars to encounter sexual violence. The very nature of a physicians’ work can be considered sexually charged and emotionally taxing. Working long hours in small groups in a new, unfamiliar environment can contribute to a breakdown of social barriers [4]. Reports of female victims are much more frequent than reports of their male colleagues. Many young specialty registrars nowadays are women, while most supervising physicians are still male. Furthermore, sexual violence is often underreported out of shame, guilt or fearing retaliation from the harassers [9]. In male students, this risk is even bigger because of the taboo on the subject as well as the long-time neglect of inclusion of male participants in research into sexual violence [10].

The fear of a negative impact on grades, the quality of the education or even career opportunities discourage many medical students and specialty registrars to report sexual violence, especially in the highly competitive environment of medical training. In short, specialty registrars have a high risk of becoming a victim of sexual violence and research is needed to examine the context wherein this happens and the actions that can be taken to prevent sexual violence from happening.

Our goal is to advocate for an effective policy to prevent sexual violence during medical training in Flanders as well as to improve the knowledge of and access to support resources for victims. In this paper, we aim to assess the prevalence of various hands-off and hands-on types of sexual violence during medical education in Flanders, Belgium, and to identify the main obstacles for reporting this behaviour.

## Methods

### Study design

The design of this study is a cross sectional study.

### Study population

The study population consisted of the 10.406 undergraduate (bachelor), graduate (master) and postgraduate (ASO/HAIO; specialty registrars) students of the five Flemish medical schools in Flanders, Belgium: Vrije Universiteit Brussel (VUB), Katholieke Universiteit Leuven (KUL), University of Antwerp (UA), Ghent University (UGent) and Hasselt University (UHasselt, only undergraduates). The only inclusion criterion was being a medical student who already participated in an internship, thus all students from the second undergraduate year until the last graduate year from all 5 universities, or specialty registrars, who were enrolled at the VUB, KUL, UA or UGent were invited.

### Instrument

As is highly recommended in sexual violence research [11, 12], we designed a survey using demographic and behaviour-specific questions inquiring whether participants had been exposed to specific types of behaviour. Thereby we avoided predefining sexual violence. First, we asked the respondent’s sex, university, current education level and cumulative duration of internships up to the enquiry. The behavioural questions were based on the ‘Understanding the Mechanisms, Nature, Magnitude and Impact of Sexual violence’ (UNMENAMAIS) questionnaire of Keygnaert et al. [3] as well as the SAV-S questionnaire by Krahé et al. [13]. We focused on those types of behaviour most applicable to medical education and added context where applicable. The behaviour types investigated both hands-on and hands-off behaviour. Within hands-off behaviour we checked for forms of sexual harassment as (attempts of) inappropriate jokes or remarks, sexting and unwanted undressing (of the victim and/or assailant or taking a recording thereof). Within hands-on behaviour we inquired on (attempts of) unwanted acts of kissing, touching, oral sex and other penetrative forms of sex. Participants were asked to limit their responses to workplace-related events.

When a respondent acknowledged having experienced a type of behaviour, we asked to specify the type of assailant (student, specialty registrar, medical staff, supervisor, paramedical staff, patient or others), the frequency of this behaviour, circumstances facilitating the behaviour (hierarchical position, inability to flee, threats regarding performance results, alcohol or drugs, physical violence, others) and if somebody reacted to this behaviour. When somebody reacted to this behaviour, we further explored who reacted (the victims themselves, students, specialty registrars, medical staff, supervisors, paramedical staff, patients or others) as well as the type of reaction according to the 4 “D’s” of bystander intervention: direct action (confronting assailants with their behaviour), distraction (distracting assailants from the situation), delay (supporting victims afterwards) and delegate (reporting to higher instance) [14]. For each “D” we explored the most applicable example of this behaviour within the context of medical education.

After looking into these specific types of behaviour, we asked respondents who fell victim to one or more behaviour types, if they had talked about what had happened to them to somebody and if so with whom: a friend or family member, a trust person (mentor, support person, faculty member, ...), a care facility or if they filed a formal complaint.

General questions about the knowledge regarding types of bystander reactions, types of victim reactions and support services in case of sexual violence were asked to all respondents at the end of the survey.

This survey consisted of a maximum of 69 questions, depending on the answers of the interviewee, and took 10 min to complete.

Before sending out the survey we ran a pilot test with a varied group of 30 participants with different profiles to screen the survey for possible bias and confusion.

### Procedure

This study was initiated and coordinated by the Vlaams Geneeskundig StudentenOverleg (VGSO), the medical student representation organisation regrouping the five medical schools in Belgium.

An introductory text explaining the goals of the study containing a link to the online survey on Surveymonkey (SurveyMonkey Inc., San Mateo, California, USA), was sent to all undergraduate (bachelor), graduate (master) and postgraduate (ASO/HAIO; specialty registrars) students of the five Flemish medical schools: Vrije Universiteit Brussel (VUB), Katholieke Universiteit Leuven (KUL), University of Antwerp (UA), Ghent university (UGent) and Hasselt University (UHasselt, only undergraduates). For privacy reasons, the invitation containing the introductory text was sent to all potential participants by the administration of each medical school using a premade template text identical for all schools. Two reminders were sent to all potential participants with a 2-week interval. The survey was open from the 1st of April 2019 until mid-May 2019 (6 weeks in total). The survey started with an information letter and online consent form, only when participants actively opted in and consented to participate, the survey questionnaire opened.

### Data analysis

The incomplete (defined as not having answered at least one behavioural question) and disqualified (did not participate in an internship yet) responses were removed, as we only wanted to examine workplace-related behaviour. The data from SurveyMonkey was exported into SPSS. We conducted all statistical analyses using SPSS statistical software, version 25.0 (SPSS Inc., Chicago, IL., USA). A chi-square test of goodness-of-fit was performed to determine whether the human sex ratio was equally obtained for the study population in comparison with the initial target population. Statistical significance was set at a *p*-value ≤0.05. Furthermore, we used descriptive statistics to quantitatively describe the differences in frequency of all variables.

### Ethics board approval

This study was approved after review of the study protocol and survey contents by the ethical board of the UZ Brussel university hospital on 18-03-2019 with the B.U.N. 143,201,837,964.

## Results

### Demographics

In total, we received 3299 responses to our survey. We firstly excluded 220 participants who had not yet participated in any internship and 64 participants because they had not answered any behaviour-specific question. Hence, 3015 valid responses were analysed (Table 1). We obtained a total response rate of 29,6% of which 35% of participants were male and 65% were female.

**Table 1:** Survey population characteristics

	*N*	*N (%)*	*n*	*n (%)*	Response rate *(% n* of *N)*
Total	10.406	*100*	3.015	*100*	*30*
Gender					
Male	4377	*42*	1048	*35*	
Female	6029	*58*	1947	*65*	
Intersexual	–	*–*	4	*0*	
Missing	–	*–*	16	*1*	
University					
KU Leuven	4812	*46*	1529	*51*	*32*
Vrije Universiteit Brussel	815	*8*	174	*6*	*21*
UGent	3070	*30*	847	*28*	*28*
Universiteit Antwerpen	1487	*14*	393	*13*	*26*
UHasselt	222	*2*	72	*2*	*32*
Study level					
Bachelor	3329	*32*	941	*31*	*28*
Masters	3809	*37*	1060	*35*	*28*
ASO: Specialty registrar	2602	*25*	698	*23*	*27*
HAIO: GP specialty registrar	666	*6*	316	*10*	*47*

The potential target population consists of 42.1% men and 57.9% women, whereas the study population consists of 35% men and 65% women. The sex ratio in the study population was not equal to the initial target population according to a chi-square test of goodness-of-fit (*p* < 0.001).

### Incidence of sexual violence in medical students and specialty registrars

Within the total study population, 1168 of 3015 participants (38,73%) reported having been victim of at least one type of sexual violence as explored by our survey. This percentage was the highest in GP specialty registrars (53%), followed by specialty registrar (50%) and master students (39%) (Table 2). A difference is observed between the relative frequency of incidents reported in male (21%) and female (48%) respondents.

**Table 2:** Distribution of reports of sexual violence

	Yes^1^		No	
	*n*	*%*	*n*	*%*
Total	1168	*39*	1847	*61*
Gender				
Male	221	*21*	827	*79*
Female	938	*48*	1009	*52*
Study level				
Bachelor	237	*25*	704	*75*
Masters	415	*39*	645	*61*
ASO: specialty registrar	349	*50*	349	*50*
HAIO: GP specialty registrar	167	*53*	149	*47*

^1^ number of participants reporting at least 1 type of behaviour defined as sexual violence, % = percentage of participants responding yes or no in each category

#### Hands-off sexual violence

Of the respondents, 32.9% (*n* = 153 men, *n* = 831 women) reported at least one experience of comments on physical appearance or disrespectful (derogatory, belittling) jokes. Patients (54.4%), medical staff members (46.6%), (fellow) students (26.7%), supervisors (25.5%) and paramedic personnel (20.1%) were identified as assailants (Table 3). The hierarchical position of the specific person exhibiting the behaviour (27.7%) and the inability to flee from the situation (20%) were most 158 frequently reported as circumstances facilitating this behaviour.

**Table 3:** Number of respondents reporting each type of hands-off sexual violence being committed by each assailant

	Total	Student		Specialty registrars		Medical staff		Supervisor		Paramedical staff		Patient		Others	
	*N*	*N*	%	*N*	%	*N*	%	*N*	%	*N*	%	*N*	%	*N*	%
Inappropriate remarks	992	94	9	171	17	462	47	253	26	199	20	540	54	47	5
Sexting	145	31	21	13	9	18	12	5	3	14	10	21	14	38	26
Remove clothes	42	7	17	2	5	7	17	1	2	0	0	23	55	3	7

Five percent (*n* = 48 men, *n* = 95 women) of the respondents reported having received unwanted sexually tinted texts or images. Moreover, being asked to undress (with or without images being taken) or (being witness to) unwarranted undressing was reported by 1.5% (*n* = 9 men, *n* = 32 women) of the respondents. Patients were cited as assailant in 55% of these cases. In 28.6% of cases this happened more than once.

#### Hands-on sexual violence

Of the respondents, 3.7% (*n* = 30 men, *n* = 73 women) had experienced an unwanted attempt to kiss. In 21% of the cases this happened more than once. Moreover, 8.7% (*n* = 44 men, *n* = 203 women) were touched or somebody tried to touch them without consent. In 39% of cases this happened more than once and in 4% this happened regularly.

There were 10 reports (*n* = 4 men, *n* = 5 women, *n* = 1 unknown) of (an attempt at) unsolicited oral sex (passive or active). The inability to escape from the situation (*n* = 1), use of alcohol or drugs (*n* = 2) and physical violence (*n* = 1) were cited as circumstances facilitating this behaviour.

There were nine reports (*n* = 3 men, *n* = 5 women, *n* = 1 unknown) of (an attempt at) unwanted sexual penetration. The (hierarchical) position of the person exhibiting the behaviour (*n* = 1), the inability to flee from the situation (*n* = 3), use of alcohol or drugs (*n* = 2) and physical violence (*n* = 1) were cited by these victims. Of the participants, two male respondents reported both unwanted oral sex and penetration.

The percentage of type of assailants of this behaviour can be found in Table 4.

**Table 4:** Number of respondents reporting each type of hands-on sexual violence being committed by each assailant

	Total	Student		Specialty registrar		Medical staff		Supervisor		Paramedical staff		Patient		Others	
	*N*	*N*	%	*N*	%	*N*	%	*N*	%	*N*	%	*N*	%	*N*	%
Kissing	105	44	42	9	9	12	11	3	3	5	5	17	16	13	12
Touching	249	34	14	27	11	87	35	33	13	24	10	80	32	16	6
Oral sex	10	1	10	0	0	0	0	0	0	1	10	1	10	4	40
Vaginal/anal penetration	9	5	56	0	0	1	11	0	0	1	11	0	0	1	11

^1^ unknown; ^2^ unknown

### Differences in incidence of sexual violence between men and women

Women reported significantly more (*p* < 0.001 and OR = 4.36) instances of sexist remarks or jokes compared to men. They also reported significantly more (*p* < 0.001 and OR = 2.69) unwanted (attempts at) physical contact. For other types of sexual violence, no significant differences between frequencies of incidents reported by men and women were observed.

### Immediate reaction to sexual violence

In most types of sexual violence, no immediate reaction followed the unwanted behaviour. Except for kissing (53%), the amount of cases in which somebody reacted was inferior to 40%, ranging from around 37% in case of unwanted touching or undressing to barely 22% in the case of unwanted penetration.

In most cases (> 80%) this reaction was by the victims themselves. Other persons were more likely to react to inappropriate remarks (45% of reactions, most often by paramedic staff, specialty registrars and supervisor) and unwanted touching (35% of reactions, most often by students, medical staff or paramedic staff). In cases of unwanted oral sex or penetration, there never was a reaction of somebody else than the victim.

**Fig. 1:**
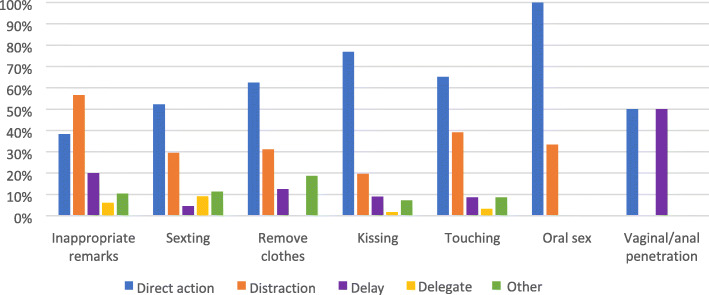
Type of response to sexual violence in the cases where somebody reacted

The type of reaction, categorised according to the 4 “D’s” of bystander reactions (direct action, distraction, delay and delegation), differed from behaviour to behaviour (Fig. 1). Direct reaction and distraction were the strategies used most often. Not many incidents were reported to higher instances. There were no statistically significant differences observed in the amount or type of reactions to sexual violence according to the assailant categories.

#### Disclosure of sexual violence

After having experienced sexual violence, women (57.3%) more often than men (39.7%) disclosed what happened. Most often, victims talked about what happened to a friend or family (92,21%). In 15% of cases they disclosed to a trust person outside of their inner circle. In 12 cases (2,5%) professional help was consulted and in 7 cases (1,5%) the authorities were notified of the event.

### Indirect exposure to sexual violence

More than one tenth (12.8%, *n* = 386) of the study population witnessed potential sexual violence towards another medical student or specialty registrar. In 56% of cases this behaviour was by a medical staff member (*n* = 215) and in 22% of cases this behaviour was by a supervisor (*n* = 83). Other reports involved patients (18%), specialty registrars (18%), paramedical staff (12%) and students (10%) as assailant.

After witnessing potential sexual violence, 48.7% asked about the feelings of the person who underwent the situation afterwards, almost one third (33.7%) undertook no action, 27.7% tried to distract the assailant, 5.9% took direct action by intervening or calling out the behaviour and 3.4% notified a superior.

### Knowledge of support mechanisms and resources in case of sexual violence

Only 40% of respondents claims to know what to do when they experience sexual violence. However, 60% of respondents indicate not to know how to handle these matters. Moreover, 62.8% of respondents do not know how to help somebody who tells them that they have experienced undesirable behaviour, nor know which instances are able to help or give support in this situation (65.1%).

## Discussion

In this study, nearly 2 out of 5 respondents reported having been victim of at least one type of sexual violence as explored by our survey. This indicates that sexual violence in medical students and postgraduates is a relatively frequent problem. Respondents most frequently complained about comments on physical appearance or disrespectful jokes. In both hands-on and hands-off sexual violence, the assailants were most frequently students, patients and medical staff. The hierarchical position of the assailant and the inability to flee from the situation are two of the most reported facilitating circumstances for sexual violence to take place. This confirms that the competitive atmosphere during medical training makes medical students and specialty registrars more susceptible to sexual violence and the hierarchical structure of a hospital scares them to speak up. These results are similar to the results of a study in Dutch medical students [15]. However, the incidence is lower than in other countries, for example in the US, with a prevalence of 33,3% in medical students and 36,2% in residents [6]. The European study of Krahé et al. shows that the prevalence in medical students is higher compared to other young adults (aged 18–27) [16].

There is a relatively large amount of reports of sexual violence by patients. Medical practitioners (including students) frequently have physical contact with patients and need to cross personal boundaries, putting them at risk of sexual violence. Sexual violence by patients was seen in a study in the Netherlands as well, where two thirds of incidents of sexual violence against medical students concerned patients [17]. Other reports involving physicians confirm sexual violence by patients is a frequent problem [18, 19]. Medical professionals focus on how to make the patient better without questioning a patient’s behaviour. Speaking up against undesired and inappropriate behaviour exhibited by patients is not a routine part of medical education. Physicians and specialty registrars must learn to accept that patients, like all people, are sexual individuals, and certain behaviour can be a manifestation of this. However, there is a definite line between acceptable and non-acceptable behaviour. These boundaries are now taught by experience, which might make them difficult to apply for students and postgraduates at the beginning of their careers. Thereby, the limited power of medical students and specialty registrars and the fear for their supervisors also plays an important role in reporting sexual violence performed by patients. Direct reaction to the patients behaviour could also influence further therapeutic relation, making it harder to respond.

Because this study did not question the study level of the respondents at the time of the reported behaviour, we cannot compare the incidence of sexual violence between undergraduate, graduate and postgraduate students. It is possible that the respondents reported events that happened earlier in their training. This is also reflected by an increase in reports as students advance in their careers and are more exposed to the workplace.

GP specialty registrars reported the highest percentage of sexual violence. This might be explained because of the isolated and private relationship they have with their supervisor working at a GP office. Considering that specialist postgraduate training is double the length of GP postgraduate training, this difference is even more remarkable.

Women reported significantly more incidents of sexist remarks or jokes and unwanted physical contact. The approximate 2:1 ratio in female:male reports was seen by Krahé et al. as well in another study in Belgium [16]. It appears in medical students this difference is even bigger compared to the Krahé paper (48% vs 21%).

In only 20–30% of cases of sexual violence, someone reacted immediately to the event. This reaction predominantly came from the victims themselves and consisted of a direct action or distraction. This might be because most people do not know how to react, which is confirmed in this study. It is also possible that bystanders are scared to respond to the behaviour, because of the position of the assailant or because of peer pressure. When somebody other than the victim responded, it mostly regarded indirect action, by asking how the victim felt after the event. In the cases of unwanted oral sex or penetration, no immediate reaction was reported by somebody else. This could be explained by the fact that the victim was alone with the assailant at that moment. It should be noted as well that most types of behaviour did not receive any reaction at all. After experiencing sexual violence, about half of the victims talked about the event to someone else, mostly friends or family. This can be of importance because these persons also stimulated the victim to report the incident, whilst the victim might not have done this if they had not disclosed.

More than one tenth of the respondents witnessed sexual violence of another medical student or colleague. Two thirds of these persons reacted to this behaviour, mostly by asking about the feelings of the person who underwent the situation, but one third undertook no action. It is possible that there is a retention bias, where respondents have forgotten or do not want to remember those occurrences of sexual violence where they did not react.

More than two thirds of respondents acknowledges not to know what to do when experiencing sexual violence personally, and 60% of respondents does not know how to react as a direct or indirect bystander nor where to get help with or to report sexual violence when they are a witness.

As this study shows sexual violence is a frequent problem in medical students and specialty registrars, it is worrisome that a lot of them (more than 50%) do not know where they can get help or report sexual violence. In Belgium, there are contact points at each medical school, governmentally sanctioning reporting points not related to medical school, as well as sexual assault care centres where victims can get holistic care and assistance upon any form after sexual violence. Unfortunately, these instances are poorly known by students and postgraduates. A first step to fight sexual violence, is to make students more aware of their existence and what assistance they can offer. Secondly, their functioning must be screened for weak points and possible issues such as lack of accessibility for medical students and specialty registrars.

In addition, in one third of the sexual violence incidents, nobody reacted and most respondents indicate not to know how to react when witnessing sexual violence. Bystander roles have proven to be an effective source of primary, secondary and tertiary prevention [20–22]. Therefore, the subject of sexual violence and bystander prevention should be addressed in the basic curricula of all medical students and all university and hospital personnel should also receive a training in taking up active bystander roles. Possible ways to do so, could be interactive trainings and general sensitisation campaigns ensuring that people know how to respond and that such behaviour is not tolerated. Furthermore, codes of conduct could be dressed for all parties involved. All of these could contribute to lowering the incidence of this behaviour and more bystander intervention when it occurs.

Notwithstanding the results, our study has several limitations that should be mentioned. The total response rate of our survey within the target population is 29,8%. This target population consists of all undergraduate, graduate and postgraduate medical students. However, our goal was to focus on internship-related sexual violence. Therefore, those (undergraduate) students indicating that they had not participated in any internship yet were disqualified. This means that the target population consists of both potential respondents as well as disqualifying respondents. It is impossible to calculate the exact target population, although we can presume this comes down to about one third of the bachelor students (first year students have no internship experience). Therefore, the real response rate is slightly higher. There is an inherent possibility of selection bias as well: possibly the respondents are the ones that have been the victim of sexual violence and have a need to talk about it. However, there is also the possibility of the opposite case where participants that have been victim of sexual violence will not respond to the survey because they feel ashamed or don’t want to relive their trauma. Some of the missing answers might be explained by respondents who experienced sexual violence and stopped completing the questionnaire because of the emotions that were raised whilst responding. Another weakness of our study is that the questionnaire was distributed by the faculty administration, which might have a deterring effect on possible respondents.

This study did not further investigate the detailed circumstances in which the sexual violence occurred. This means that behaviour exhibited by cognitively impaired patients - such as patients suffering from dementia or patients under the influence of alcohol - is not researched in this result. We wanted to focus our study on sexual violence related to the status of being a medical student, but this can be interpreted broadly. A lot of sexual violence is not workplace-related however, and this will not be detected by our study. However, we did observe 25% of undergraduate students reporting workplace related sexual violence which is remarkably high with regards to their limited internship experience (less than 1 month).

For future research, it is important to keep monitoring the prevalence of sexual violence in this population, to keep track of the evolution and to further investigate in which context sexual violence occurs. Doing this, better tools can be developed to prevent such behaviour. It would be interesting to investigate the prevalence of sexual violence in other healthcare workers and students, such as nursing students, to see if sexual violence is a problem in these groups as well.

## Conclusion

This study shows that sexual violence is an important issue in medical students and specialty registrars in Flanders. Thereby, most of respondents acknowledged not to know how to respond to this kind of behaviour*.* This means that medical trainees and the people surrounding them need more sensitization and training about sexual violence and the bystander roles, to lower the incidence of sexual violence

## Data Availability

The datasets used and/or analysed during the current study are available from the corresponding author on reasonable request.

## Abbreviations

GP: General practitioner
ASO: Arts-specialist in opleiding, specialty registrar
HAIO: Huisarts in opleiding, GP specialty registrar
VUB: Vrije Universiteit Brussel
KUL: Katholieke Universiteit Leuven
UA: University of Antwerp
UGent: Ghent University
UHasselt: Hasselt University
VGSO: Vlaams Geneeskundig StudentenOverleg
